# Tonic inhibition of murine proximal colon is due to nitrergic suppression of Ca^2+^ signaling in interstitial cells of Cajal

**DOI:** 10.1038/s41598-019-39729-7

**Published:** 2019-03-13

**Authors:** Bernard T. Drumm, Benjamin E. Rembetski, Salah A. Baker, Kenton M. Sanders

**Affiliations:** Department of Physiology & Cell Biology, University of Nevada, Reno School of Medicine, Reno, NV 89557 USA

## Abstract

Spontaneous excitability and contractions of colonic smooth muscle cells (SMCs) are normally suppressed by inputs from inhibitory motor neurons, a behavior known as tonic inhibition. The post-junctional cell(s) mediating tonic inhibition have not been elucidated. We investigated the post-junctional cells mediating tonic inhibition in the proximal colon and whether tonic inhibition results from suppression of the activity of Ano1 channels, which are expressed exclusively in interstitial cells of Cajal (ICC). We found that tetrodotoxin (TTX), an inhibitor of nitric oxide (NO) synthesis, L-NNA, and an inhibitor of soluble guanylyl cyclase, ODQ, greatly enhanced colonic contractions. Ano1 antagonists, benzbromarone and Ani9 inhibited the effects of TTX, L-NNA and ODQ. Ano1 channels are activated by Ca^2+^ release from the endoplasmic reticulum (ER) in ICC, and blocking Ca^2+^ release with a SERCA inhibitor (thapsigargin) or a store-operated Ca^2+^ entry blocker (GSK 7975 A) reversed the effects of TTX, L-NNA and ODQ. Ca^2+^ imaging revealed that TTX, L-NNA and ODQ increased Ca^2+^ transient firing in colonic ICC. Our results suggest that tonic inhibition in the proximal colon occurs through suppression of Ca^2+^ release events in ICC. Suppression of Ca^2+^ release in ICC limits the open probability of Ano1 channels, reducing the excitability of electrically-coupled SMCs.

## Introduction

Contractions of the smooth muscle cells (SMCs) in the proximal colon are essential for colonic motility that assists in reabsorption of water and electrolytes and eventually propels fecal materials toward the distal colon and rectum. Contractions of the proximal colon are regulated by intrinsic and extrinsic motor neurons, but neural controls are superimposed upon myogenic mechanisms that set the excitability of SMCs. The term ‘myogenic’, once exclusive to the cellular mechanisms of SMCs, now includes mechanisms attributed to interstitial cells, such as interstitial cells of Cajal (ICC) and platelet-derived-growth-factor-receptor-alpha^+^ (PDGFRα^+^) cells. Together these cells make up a complex of electrically-coupled cells, known collectively as the SIP syncytium^1,2^.

ICC regulate gastrointestinal (GI) motility through Ca^2+^ entry and release events that activate Ca^2+^-activated Cl^−^ channels encoded by *Ano1*^3–6^. Ano1 mediates inward currents, and activation or suppression of these events, affects the basal excitability of SMCs. Activation of Ano1 channels is responsible for the pacemaker events that generate propagating electrical slow waves, and these channels are also involved in responses to some enteric motor neurotransmitters^7,8^. In contrast, PDGFRα^+^ cells regulate colonic motility by exerting a hyperpolarizing effect on the SIP syncytium through the activation of small conductance Ca^2+^-activated K^+^ (SK) channels^9–13^.

Colonic SMCs are intrinsically active spontaneously due to their resting potentials that are at or near the threshold for activation of voltage-dependent Ca^2+^ channels. If left alone SMCs generate action potentials, but action potentials are unable to propagate over great distances in smooth muscle syncytia and entrain the behaviors of other cells^14^. Therefore, the intrinsic excitability of SMCs on its own generates localized excitation and fibrillatory- like contractile behavior that is non-productive for propulsion of luminal contents. To generate orderly and propulsive contractions, the activity of SMCs must be suppressed so inputs from ICC and motor neurons can coordinate contractions. Suppression of SMC excitability is known as ‘tonic inhibition’, a major form of motility behavior in muscles of the distal GI tract and recognized for decades^15^. Tonic inhibition in colonic muscles results from release of inhibitory neurotransmitters. Tetrodotoxin (TTX) or nitric oxide (NO) synthase inhibitors, such as *N*_ω_-Nitro-L-arginine (L-NNA) increase the excitability of colonic muscles from mouse^16–18^, rat^19–22^, cat^23^, canine^24^ and human^25,26^. Tonic inhibition leads to relative electrical and mechanical quiescence between colonic migrating motor complexes (CMMCs), and muscle excitability is thought to increase due to suppression of inhibitory neurons and firing of excitatory neurons at the onset of the CMMC^27^. Impairment of tonic inhibition has been linked to conditions such as chronic constipation and the pseudo-obstruction that occurs in infants with Hirschprungs disease^28^. However, the post-junctional cell(s) and intracellular mechanisms responsible for linking neural input to tonic inhibition are not well understood.

ICC in the colon generate spontaneous transient inward currents (STICs) due to expression of Ca^2+^-activated Cl^−^ channel encoded by *Ano1*^3,29^. Ca^2+^ transients in ICC, due to release from intracellular Ca^2+^ stores, are coupled to activation of Ano1^4,6,30^ and we found recently that NO suppresses Ca^2+^ transients in ICC from the small intestine^31,32^. Ano1 channels are expressed exclusively in ICC in GI muscles^3,29^. Therefore, we investigated whether tonic inhibition, due to nitrergic mechanisms in the proximal colon, occurs as a result of mechanisms present in ICC. Results are presented showing that nitrergic inputs suppress Ca^2+^ release events in ICC in the proximal colon, and the enhancement in contractions elicited by blocking tonic inhibition are suppressed by Ano1 channel antagonists.

## Results

**Figure 1:**
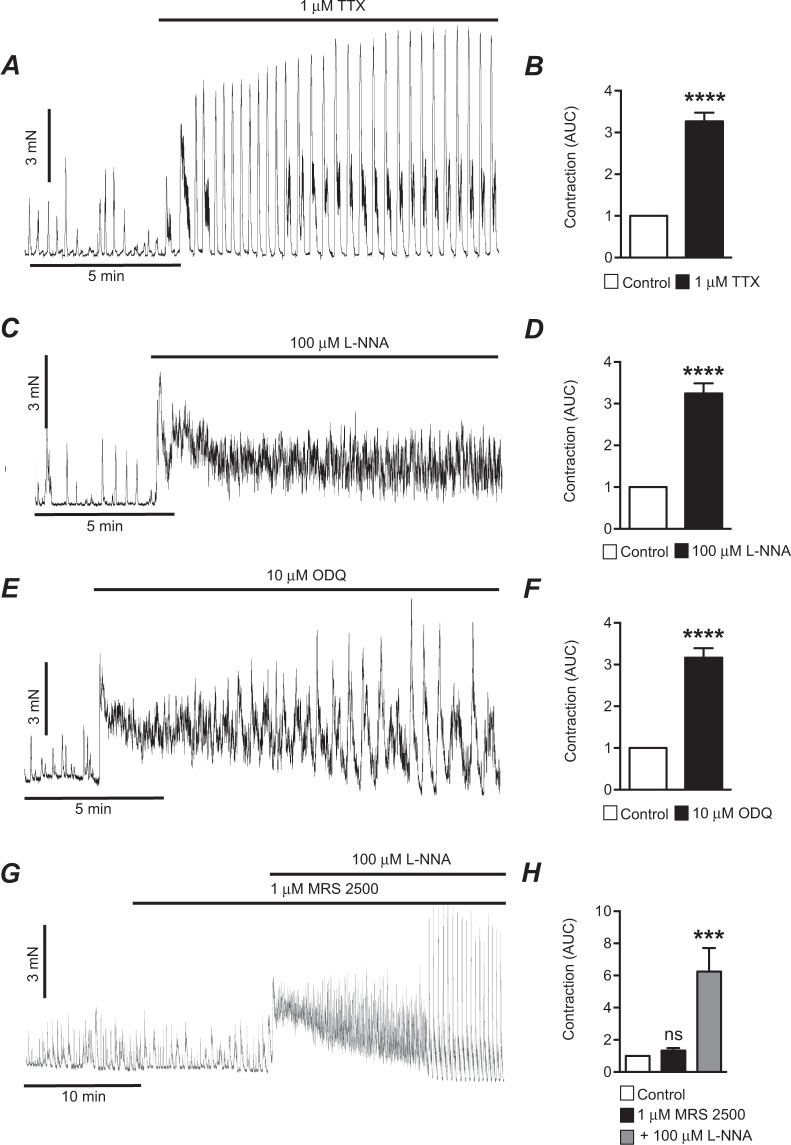
Tonic inhibition in circular muscle of mouse proximal colon. (**A**) Representative contractile trace showing the effects of blocking input from enteric nerves with TTX (1 μM) on contractions of mouse proximal colon. (**B**) Summarized data showing the enhancement in contractions (AUC) caused by TTX (1 μM) in the mouse proximal colon (n = 70). (**C**) Representative contractile trace showing the effects of blocking NO synthesis with L-NNA (100 μM) on contractions of mouse proximal colon. (**D**) Summarized data showing the enhancement of contractions (AUC) after adding L-NNA (100 μM; n = 61). (**E**) Representative contractile trace showing the effects the sGC inhibitor, ODQ (10 μM), on contractions of the mouse proximal colon. (**F**) Summarized data showing the enhancement of contractions (AUC) after adding ODQ (10 μM; n = 64). (**G**) Representative contractile trace showing the effects of a P_2_Y_1_ antagonist, MRS2500 (1 μM), on contractions of the mouse proximal colon. The P_2_Y_1_ antagonist had minimal effects on contraction. Subsequent addition of L-NNA (100 μM) enhanced contractions significantly. (**H**) Summarized data showing the effects of MRS2500 (1 μM) and subsequent addition of L-NNA (100 μM) on contractions (n = 12). ns = P > 0.05, ***P < 0.001, ****P < 0.0001.

Murine proximal colon muscles exhibited spontaneous phasic contractions (Fig. 1). Under basal conditions, phasic contractions were restrained by tonic inhibitory input from enteric neurons, because addition of TTX (1 μM) caused dramatic increases in contractions (Fig. 1A). Normalized AUC increased 3.3 ± 0.2 fold in response to TTX (Fig. 1B, P < 0.0001, n = 70). The response to TTX was characterized by either a marked increase in sustained contraction before a large increase in the magnitude and duration of phasic contractions occurred or as an increase in frequency and magnitude without an initial increase in tone. The causes for these differences in initial responses were not explored. The excitatory effects of TTX were mimicked by an inhibitor of nitric oxide synthesis, L-NNA (100 μM, Fig. 1C). Similar to the responses to TTX, L-NNA caused either an increase in the firing frequency and magnitude of contractions or elicited a sustained increase in colonic tone, as illustrated in Fig. 1C. AUC of colonic contractions increased 3.2 ± 0.2 fold after application of L-NNA (Fig. 1D, P < 0.0001, n = 61).

In the proximal colon, NO exerts its inhibitory effects via activation of soluble guanylate cyclase (sGC) and generation of cyclic guanosine monophosphate (cGMP)^33^. We tested an inhibitor of sGC, 1*H*-[1,2,4] Oxadiazolo [4,3-*a*] quinoxalin-1-one (ODQ), to determine whether blocking this signaling pathway had the same effects as TTX and L-NNA. ODQ (10 μM) increased AUC 3.2 ± 0.2 fold (Fig. 1E,F, P < 0.0001, n = 64). In contrast to the effects of blocking NO synthesis and transduction, the purinergic receptor (P_2_Y_1_) antagonist (MRS 2500, 1 μM) did not cause an appreciable increase in proximal colon contractions (Fig. 1G,H, P > 0.05, n = 12). When L-NNA was applied to the same muscles treated with MRS2500, marked increases in contractions occurred (Fig. 1H, P < 0.001, n = 12).

**Figure 2:**
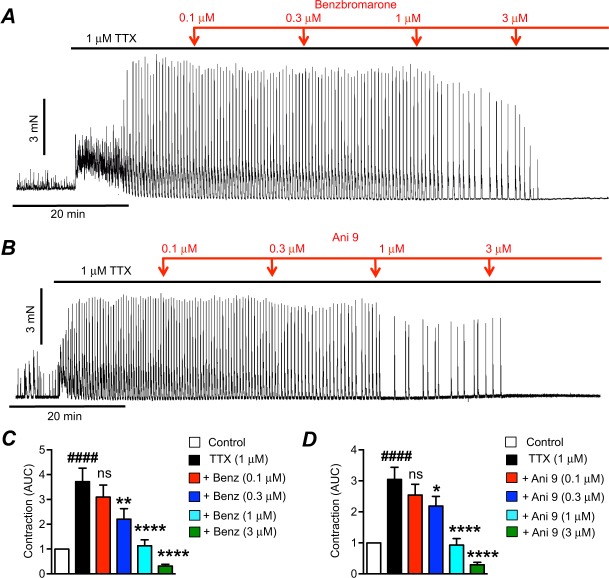
The effects of Ano1 channel antagonists on the enhanced contractions after TTX. (**A**) Representative contractile trace showing the effects of benzbromarone (0.1–3 μM) on contractions after TTX (1 μM). (**B**) Representative contractile trace showing the effects of Ani 9 (0.1–3 μM) on contractions after TTX (1 μM). (**C**) Summarized data showing the effects of benzbromarone (0.1–3 μM) on contractions after TTX (1 μM; n = 13). (**D**) Summarized data showing the effects of Ani 9 (0.1–3 μM) on contractions after TTX (1 μM; n = 8). ^####^P < 0.0001 compared to control, ns = P > 0.05 compared to TTX, *P < 0.05 compared to TTX, **P < 0.01 compared to TTX, ****P < 0.0001 compared to TTX.

After tonic inhibition was relieved by addition of TTX, application of Ano1 inhibitors^34–36^ benzbromarone (Fig. 2A, 0.1–3 μM) or Ani 9 (Fig. 2B, 0.1–3 μM) inhibited contractions of colonic muscle. At a concentration of 0.3 μM, benzbromarone significantly reduced AUC from 3.7 ± 0.5 to 2.2 ± 0.4 (Fig. 2C, P < 0.01, n = 13). AUC was further reduced to 1.1 ± 0.2 and 0.3 ± 0.07 by 1 μM and 3 μM benzbromarone, respectively (Fig. 2C, P < 0.0001, n = 13). Ani 9 reduced AUC from 3 ± 0.4 to 2.2 ± 0.3 (0.3 μM Ani 9, Fig. 2D, P < 0.05, n = 8), 0.9 ± 0.2 (1 μM Ani 9, Fig. 2D, P < 0.0001, n = 8), and 0.3 ± 0.08 (3 μM Ani 9, Fig. 2D, P < 0.0001, n = 8). The Ano1 antagonists were given in cumulative doses in this portion of the study, and this required muscles to be exposed to TTX continuously for 90–100 mins. Therefore, we performed time controls with equivalent periods of TTX exposure to be sure that the responses to Ano1 antagonists were not simply due to rundown of contractions. The increase in contractions and AUC was sustained for the periods of time required for exposure to the full range of Ano1 antagonists (Fig. S1A). Quantitative measurements of AUC every 20 mins in the presence of TTX showed no rundown of contractions over the time course of the 100 min exposures (Fig. S1A,C, P > 0.05, n = 9).

Ano1 antagonists required solubilization in DMSO. Therefore, DMSO vehicle control experiments were also performed using the highest concentration of DMSO (0.03%) used in the study of Ano1 antagonists. Long-term application (up to 60 min) of DMSO (0.03%) to muscles after TTX had no significant effects on colonic contractions (Fig. S1B,D, P > 0.05, n = 9). These experiments showed that the effects of Ano1 inhibitors were not due to time rundown or vehicle effects.

**Figure 3:**
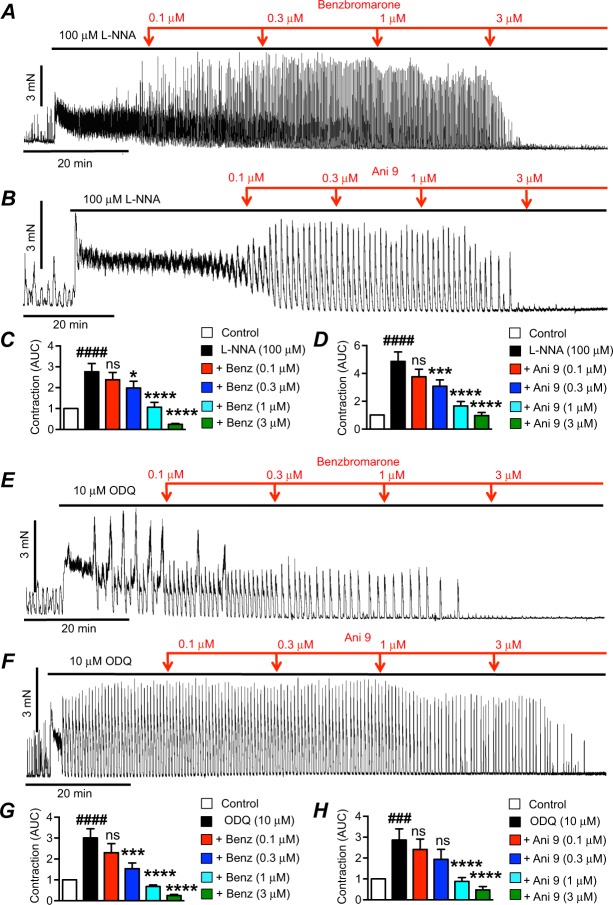
Effects of Ano1 antagonists on contractions after L-NNA or ODQ. (**A**) Representative contractile trace showing effects of benzbromarone (0.1–3 μM) on contractions after L-NNA (100 μM). (**B**) Representative contractile trace showing effects of Ani 9 (0.1–3 μM) after L-NNA (100 μM) on contractions. (**C**) Summarized data showing the effects of benzbromarone (0.1–3 μM) on the enhanced contractions (AUC) after adding L-NNA (100 μM; n = 12). (**D**) Summarized data showing the effects of Ani 9 (0.1–3 μM) on the enhanced contractions (AUC) after adding L-NNA (100 μM; n = 8). (**E**) Representative contractile trace showing the effects of benzbromarone (0.1–3 μM) on the excitatory response of ODQ (10 μM) on contractions. (**F**) Representative contractile trace showing the effect of Ani 9 (0.1–3 μM) on the enhanced contractions (AUC) after adding ODQ (10 μM) on proximal colon contractions. (**G**) Summarized data showing the effect of benzbromarone (0.1–3 μM) on the excitatory response of ODQ (10 μM) on proximal colon contractions (n = 12). (**H**) Summarized data showing the effect of Ani 9 (0.1–3 μM) on the excitatory response of ODQ (10 μM) on proximal colon contractions (n = 7). ^###^P < 0.001 compared to control, ^####^P < 0.0001 compared to control, ns = P > 0.05 compared to TTX, *P < 0.05 compared to TTX, ***P < 0.01 compared to TTX, ****P < 0.0001 compared to TTX.

Similar to their effects on colonic contractions elicited by TTX, the Ano1 antagonists also inhibited contractions stimulated by L-NNA (Fig. 3A,B) and ODQ (Fig. 3E,F). Benzbromarone reduced the normalized AUC of contractions after L-NNA from 2.7 ± 0.4 to 2 ± 0.3 (0.3 μM Benzbromarone, Fig. 3C, P < 0.05, n = 12), 1.1 ± 0.2 (1 μM Benzbromarone, Fig. 3C, P < 0.0001, n = 12) and 0.2 ± 0.05 (3 μM Benzbromarone, Fig. 3C, P < 0.0001, n = 12). Ani 9 reduced AUC after L-NNA from 4.9 ± 0.7 to 3.1 ± 0.5 (0.3 μM Ani 9, Fig. 3D, P < 0.001, n = 8), 1.7 ± 0.3 (1 μM Ani 9, Fig. 3D, P < 0.0001, n = 8) and 1.0 ± 0.2 (3 μM Ani 9, Fig. 3D, P < 0.0001, n = 8). In the presence of ODQ, AUC was reduced by benzbromarone from 3 ± 0.4 normalized AUC to 1.5 ± 0.3 (0.3 μM Benzbromarone, Fig. 3G, P < 0.001, n = 12), 0.7 ± 0.07 (1 μM Benzbromarone, Fig. 3G, P < 0.0001, n = 12), and 0.25 ± 0.05 (3 μM Benzbromarone, Fig. 3G, P < 0.0001, n = 12). Ani 9 reduced ODQ induced increases in contraction from 2.9 ± 0.5 normalized AUC to 0.9 ± 0.2 and 0.5 ± 0.2 normalized AUC by 1 and 3 μM Ani9 respectively (Fig. 3H, P < 0.0001, n = 7).

**Figure 4:**
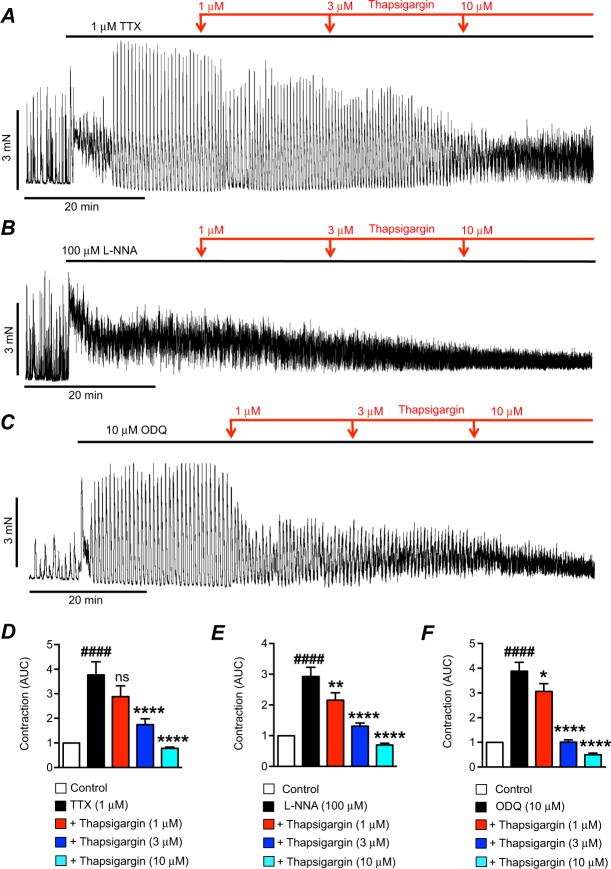
Effects of thapsigargin on contractions after TTX, L-NNA or ODQ. (**A**) Representative trace showing the effects of thapsigargin (1–10 μM) on the contractions of proximal colon after TTX (1 μM). (**B**) Representative trace showing the effects of thapsigargin (1–10 μM) on the contractions of proximal colon after L-NNA (100 μM). (**C**) Representative trace showing the effects of thapsigargin (1–10 μM) on the contractions of proximal colon after ODQ (10 μM). (**D**) Summarized data showing the effects of thapsigargin (1–10 μM) on contractions after TTX (1 μM; n = 15). (**E**) Summarized data showing the effects of thapsigargin (1–10 μM) on contractions after L-NNA (100 μM; n = 14). (**F**) Summarized data showing the effects of thapsigargin (1–10 μM) on contractions after ODQ (10 μM; n = 13). ^####^P < 0.0001 compared to control, ns = P > 0.05 compared to TTX, *P < 0.05 compared to TTX, **P < 0.01 compared to TTX, ****P < 0.0001 compared to TTX.

Ano1 is expressed exclusively in ICC in GI muscles^29^, and its activation is due to Ca^2+^ release in these cells^6^. Ca^2+^ release events in ICC are mediated by release of Ca^2+^ from endoplasmic reticulum (ER) stores via Ca^2+^ channels such as inositol-tri-phosphate receptors (IP_3_Rs) and ryanodine receptors (RyRs). Sustained Ca^2+^ release from stores requires refilling by the sarco/endoplasmic-reticulum-Ca^2+^-ATPase (SERCA) pump^30,31,37^. Therefore, we tested the effects of the SERCA pump inhibitor thapsigargin (1–10 μM) on the excitatory effects of TTX, L-NNA and ODQ. Thapsigargin reduced the excitatory effects induced by TTX (Fig. 4A), L-NNA (Fig. 4B) and ODQ (Fig. 4C). In the presence of TTX, thapsigargin caused significant reduction in contractions at 3 μM, reducing the normalized AUC of colonic contractions in TTX from 3.8 ± 0.5 to 1.7 ± 0.2 (Fig. 4D, P < 0.0001, n = 15). AUC was further reduced to 0.8 ± 0.05 by 10 μM thapsigargin (Fig. 4D, P < 0.0001, n = 15). Thapsigargin also reduced the AUC of contractions in the presence of L-NNA, reducing AUC from 2.9 ± 0.3 to 2.2 ± 0.2 (1 μM Thapsigargin, Fig. 4E, P < 0.01, n = 14), 1.3 ± 0.1 (3 μM Thapsigargin, Fig. 4E, P < 0.0001, n = 14), and 0.7 ± 0.06 (10 μM Thapsigargin, Fig. 4E, P < 0.0001, n = 14). Inhibitory effects on the ODQ response were also observed with 1 μM thapsigargin. AUC was reduced from 3.9 ± 0.4 to 3.1 ± 0.3 (Fig. 4F, P < 0.05, n = 13), and to 1 ± 0.1 (Fig. 4F, P < 0.0001, n = 13) and 0.5 ± 0.06 (Fig. 4F, P < 0.0001, n = 13) by 3 μM and 10 μM thapsigargin, respectively.

**Figure 5:**
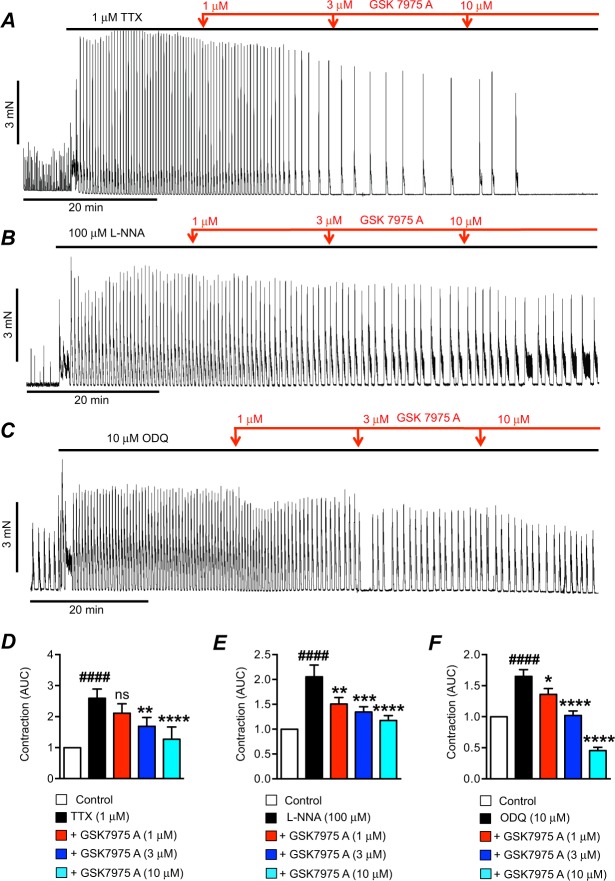
Effects of GSK 7975 A on contractions after TTX, L-NNA or ODQ. (**A**) Representative trace showing the effects of GSK 7975 A (1–10 μM) on the contractions of proximal colon after TTX (1 μM). (**B**) Representative trace showing the effects of GSK 7975 A (1–10 μM) on the contractions of proximal colon after L-NNA (100 μM). (**C**) Representative trace showing the effects of GSK 7975 A (1–10 μM) on the contractions of proximal colon after ODQ (10 μM). (**D**) Summarized data showing the effects of GSK 7975 A (1–10 μM) on contractions after TTX (1 μM; n = 13). (**E**) Summarized data showing the effects of GSK 7975 A (1–10 μM) on contractions after L-NNA (100 μM; n = 9). (**F**) Summarized data showing the effects of GSK 7975 A (1–10 μM) on contractions after ODQ (10 μM; n = 10). ^####^P < 0.0001 compared to control, ns = P > 0.05 compared to TTX, *P < 0.05 compared to TTX, **P < 0.01 compared to TTX, ***P < 0.001 compared to TTX, ****P < 0.0001 compared to TTX.

Although Ca^2+^ stores can be refilled in the short term from the cytoplasm by SERCA, sustaining Ca^2+^ release events for long periods may require Ca^2+^ recovery from the extracellular fluid, and additional refilling mechanisms may be required. Store-operated-Ca^2+^-entry (SOCE) was found previously to be important in ICC for sustaining Ca^2+^ release events and maintaining pacemaker activity in the GI muscles^38^. In ICC, SOCE appears to be mediated by interactions between the ER protein, stromal interacting molecule (STIM), and Orai, a family of Ca^2+^ influx channels in the plasma membrane^38^. We tested the effects of an Orai channel blocker (GSK 7975 A) on the contractile activity enhanced by TTX, L-NNA and ODQ. GSK 7975 A inhibited the contractions after TTX (Fig. 5A), and the same effect was observed on the responses to L-NNA (Fig. 5B) and ODQ (Fig. 5C). Application of GSK 7975 A caused an increased interval between contractions as well as a reduction in the magnitude, an effect that is consistent with a reduced refilling of intracellular Ca^2+^ stores. GSK 7975 A (3 μM) reduced the AUC of colonic contractions in the presence of TTX from 2.6 ± 0.3 to 1.7 ± 0.3 (Fig. 5D, P < 0.01, n = 13) and this was reduced to 1.3 ± 0.4 by 10 μM GSK 7975 A (Fig. 5D, P < 0.0001, n = 13). At all 3 doses tested GSK 7975 A also reduced the excitatory effects of L-NNA and ODQ. Contractions in L-NNA were reduced from a normalized AUC of 2.1 ± 0.2 to 1.5 ± 0.1 (1 μM GSK 7975 A, Fig. 5E, P < 0.01, n = 9), 1.3 ± 0.1 (3 μM GSK 7975 A, Fig. 5E, P < 0.001, n = 9), and 1.1 ± 0.1 (10 μM GSK 7975 A, Fig. 5E, P < 0.0001, n = 9). Contractions in ODQ were reduced from a normalized AUC of 1.6 ± 0.1 to 1.4 ± 0.1 (1 μM GSK 7975 A, Fig. 5F, P < 0.05, n = 10), 1 ± 0.07 (3 μM GSK 7975 A, Fig. 5F, P < 0.0001, n = 10), and 0.45 ± 0.05 (10 μM GSK 7975 A, Fig. 5F, P < 0.0001, n = 10).

Many of the protocols above used cumulative addition of increasing doses of drugs so inhibition of contractions occurred in a time-dependent manner. Some of the agents would be expected to have more immediate effects. Therefore, we tested intermediate concentrations of these agents to display the immediate effects. As shown in the examples in Supplementary Fig. 2, single sub–maximal concentrations of each agent induced rapid decreases in colonic contractions. Benzbromarone (1 μM) decreased TTX induced contractions from 2.2 ± 0.3 normalized AUC to 0.6 ± 0.1 normalized AUC (Fig. S2A,E, P < 0.0001, n = 23). Ani 9 (1 μM) decreased TTX induced contractions from 2.9 ± 0.4 to 1 ± 0.1 normalized AUC (Fig. S2B,F, P < 0.0001, n = 14). Thapsigargin (3 μM) concentration decreased TTX induced contractions from 2.6 ± 0.2 to 1.5 ± 0.2 normalized AUC (Fig. S2C,G, P < 0.0001, n = 27). GSK 7975 A (3 μM) decreased TTX induced contractions from 2.9 ± 0.6 normalized AUC to 1.1 ± 0.2 normalized AUC. (Fig. S2D,H, P < 0.01, n = 13).

**Figure 6:**
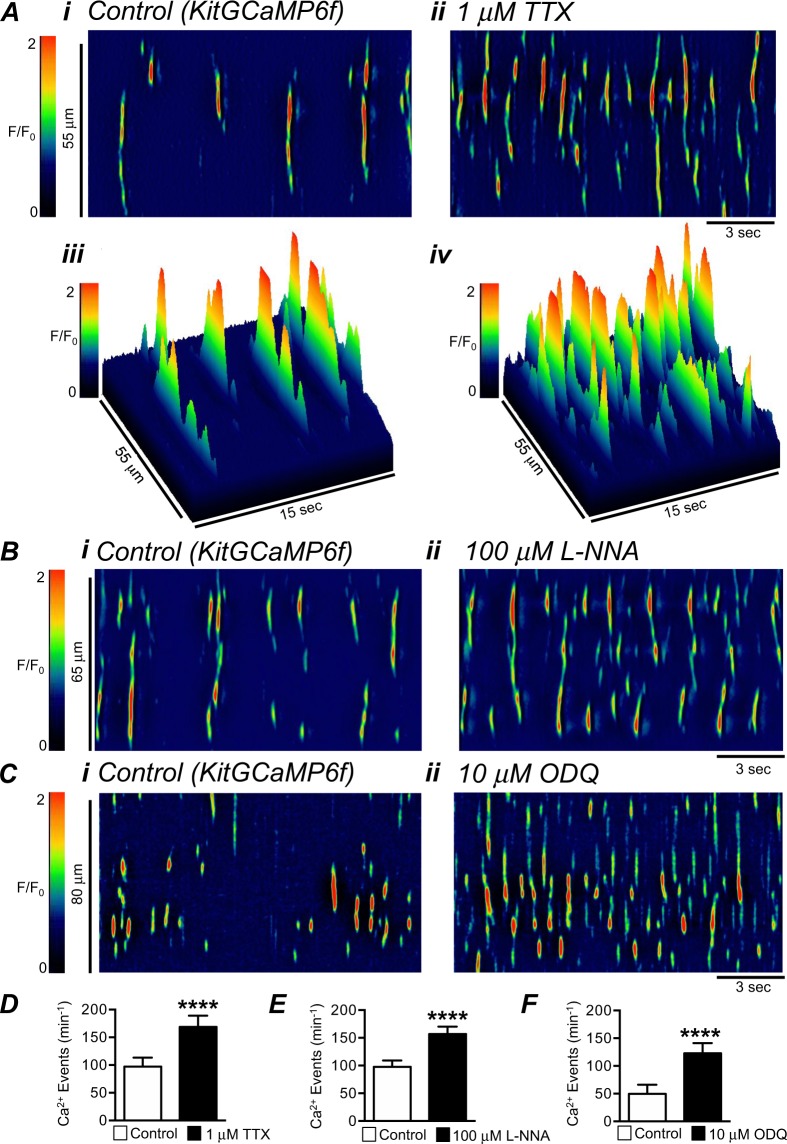
Effect of TTX, L-NNA and ODQ on Ca^2+^ transients in colonic ICC. (**A**) Representative STMs of spontaneous Ca^2+^ transients in colonic intramuscular ICC (ICC-IM) recorded *in situ* with a 60x objective in control (i) and in the presence of TTX (1 μM; ii), these maps are also displayed in 3-D format (iii–iv). (**B**) Representative STMs of spontaneous Ca^2+^ transients in ICC-IM recorded *in situ* in control (i) and in the presence of L-NNA (100 μM; ii). (**C**) Representative STMs of spontaneous Ca^2+^ transients in ICC-IM recorded *in situ* in control (i) and in the presence of ODQ (10 μM; ii). (**D**–**F**) Summarized data for the effects of TTX (n = 22), L-NNA (n = 19) and ODQ (n = 8) on spontaneous Ca^2+^ transient frequency in ICC. ****P < 0.0001 compared to control.

Our hypothesis is that Ano1 channels in GI muscles are activated when nitrergic neural regulation is relieved, and the increase in Ano1 activity is responsible for the significant increase in colonic contractions. Coupling between nitrergic inhibition and increased activation of Ano1 channels should be associated with increased Ca^2+^ release in ICC, and this was tested using colonic muscles expressing GCaMP6f in ICC (see Methods). Intramuscular ICC (ICC-IM) were imaged in these experiments, as these cells are thought to be involved in neuro-effector transduction in GI muscles^39,40^. Colonic ICC-IM generated spontaneous Ca^2+^ transients similar to those previously characterized in the ICC in the region of the deep muscular plexus in the small intestine^31^. Spatio-temporal maps (STMs) of Ca^2+^ transients recorded from ICC-IM in the proximal colon *in situ* revealed that Ca^2+^ transients arose from multiple sites along the lengths of individual ICC-IM and were stochastic in their firing patterns (Fig. 6A). TTX increased the firing frequency of Ca^2+^ transients significantly from 97 ± 16.4 min^−1^ in control to 168.8 ± 20.2 min^−1^ after addition of TTX (Fig. 6A,D, P < 0.0001, n = 22). L-NNA and ODQ also increased the firing frequency of Ca^2+^ transients in ICC-IM (Fig. 6B,C). L-NNA increased Ca^2+^ transient firing frequency from 97.9 ± 11.3 min^−1^ to 156.8 ± 13.4 min^−1^ (Fig. 6E, P < 0.0001, n = 19), and ODQ increased Ca^2+^ transient firing frequency from 49.9 ± 16.5 min^−1^ to 122.9 ± 18.4 min^−1^ (Fig. 6F, P < 0.0001, n = 8).

**Figure 7:**
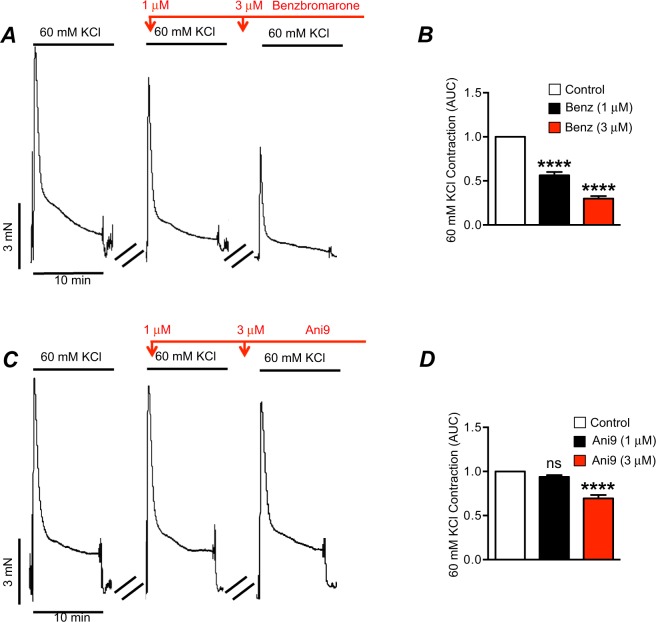
Effects of benzbromarone and Ani9 on KCl induced contractions. (**A**) Representative KCl (60 mM) induced contractions of colonic muscle in the presence of TTX (1 μM), L-NNA (100 μM) and atropine (1 μM) showing the effects of benzbromarone (1–3 μM). (**B**) Summary data for the effects of benzbromarone (1–3 μM) on contractions evoked by elevated [K^+^]_o_ (60 mM; n = 17). (**C**) Representative contractile trace of KCl (60 mM) induced contractions of colonic muscle in the presence of TTX (1 μM), L-NNA (100 μM) and atropine (1 μM) showing the effect of Ani 9 (1–3 μM). (**D**) Summary data for the effects of Ani9 (1–3 μM) on contractions evoked by Elevated [K^+^]_o_ (60 mM; n = 17). ns = P > 0.05 compared to control. ****P < 0.0001 compared to control.

We also tested an alternative explanation for our findings that Ano1 antagonists inhibit the contractions enhanced by relief of tonic inhibition because some Ano1 antagonists have been shown to block L-type Ca^2+^ currents^41^. This possibility was assessed by testing the effects of the Ano1 antagonists we used (Ani9 and Benzbromarone) on contractile responses of proximal colon muscles to elevated external K^+^ ([K^+^]_o_). These experiments were performed in the presence of TTX (1 μM), L-NNA (100 μM) and atropine (1 μM) to minimize confounding effects from depolarization-dependent release of major motor neurotransmitters. Elevated [K^+^]_o_ (60 mM) evoked reproducible contractures in colon muscle. Benzbromarone (1 μM) reduced elevated [K^+^]_o_ contractions to 56.4 ± 3.7% of control (Fig. 7B, P < 0.0001, n = 17), and 3 μM benzbromarone inhibited contractions to 30 ± 2.7% of control (Fig. 7B, P < 0.0001, n = 17). In contrast, Ani9 (1 μM) had no significant effect on elevated [K^+^]_o_ contractions (Fig. 7C,D, P > 0.05, n = 17), but effects were observed at 3 μM where Ani9 reduced contractions to 70 ± 4% of control (Fig. 7D, P < 0.0001, n = 17).

## Discussion

Tonic inhibition is a well-known motor phenomenon in GI muscles of mice^16–18^, rats^19–22^, cats^23^, dogs^24^ and humans^25,26^ that is thought to be caused by the sustained release of NO from enteric neurons. The musculature of the colon contains **S**MCs, **I**CC, and **P**DGFRα^+^ cells making up the **SIP** syncytium, and enteric neurons innervate the SIP syncytium with different responses being mediated by different cells^1^. In the current study, we investigated the post-junctional cellular targets that mediate tonic inhibition of smooth muscle contractions in the mouse proximal colon. Blocking the nitrergic pathway by blocking enteric neurons (TTX), inhibition of nNOS (L-NNA) or blocking the receptor for NO (ODQ) unleashed increases in myogenic contractions of the mouse proximal colon. Localized Ca^2+^ transients were observed in ICC-IM of the proximal colon that have been attributed to release of Ca^2+^ from stores and coupled to activation of Ano1 Cl^−^ channels in other classes of ICC^6,8,30–32^. The agents that block the nitrergic pathway greatly increased the Ca^2+^ transients in ICC-IM and increased the contractile responses of intact muscles. Ano1 channel antagonists, thapsigargin and the Orai channel blocking drug, GSK7975A, inhibited this contractile activity. Taken together, these data suggest that tonic inhibition (i.e. spontaneous nitrergic neural input) results from suppression of Ca^2+^ release events in ICC, which reduces the activation of Ano1 channels and maintains a low level of excitability in the SIP syncytium. Another interpretation of our results could be that the Ano1 antagonists block voltage-dependent Ca^2+^ channels, and this is the cause of colonic inhibition. While control experiments showed this is a concern with benzbromarone, as discussed below, significant effects on colonic contractions were observed with Ani9 at a concentration that blocks Ano1 channels^35^ and had little or no effect on elevated [K^+^]_o_ induced contractions.

**Figure 8:**
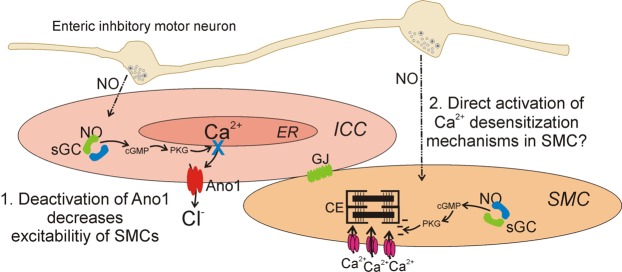
Tonic inhibition is due to effects on ICC. Schematic illustrating how tonic inhibition affects ICC. Release of nitric oxide (NO) from enteric inhibitory motor neurons activates sGC in ICC, which leads to increased cGMP, and a subsequent decrease in Ca^2+^ release from ER. Reduced Ca^2+^ release decreases Ano1 activation, which due to the electrically coupled nature of the SIP syncytium leads to reduced excitability of SMCs. Also possible, but not tested in the current study, might be direct effects of NO on SMCs that may reduce contractions via the NO-sGC-cGMP pathway, leading to desensitization of the contractile element (CE) to cytoplasmic Ca^2+^.

NO released from enteric inhibitory neurons is thought to be transduced, in part, by ICC (Fig. 8)^42^. Transduction of nitrergic inputs mediated by ICC most likely results in electrical responses (e.g. hyperpolarization due to openings of K^+^ channels or reduced activation of inward currents due to Cl^−^ or non-selective channels)^42^ that are conducted to electrically coupled SMCs in the SIP syncytium. Evidence for innervation and transduction of NO by ICC includes: (i) very close associations (<20 nm) between ICC and nitrergic nerve terminals^40,43,44^, (ii) neural inhibition of spontaneous Ca^2+^ transients in ICC that can be relieved by TTX or L-NNA^31,32,45,46^, (iii) expression of signaling molecules required for post-junctional nitrergic responses in ICC^40,43^; (iv) reduction or loss of nitrergic responses in animals with ICC-specific knockdown of nitrergic signaling molecules^47–49^.

Ano1 channels are expressed exclusively in ICC in GI muscles^29^, and activation of these channels is required for the principal functions of ICC, pacemaking and neurotransduction, because genetic ablation of *Ano1* causes loss of pacemaker activity in the stomach and small intestine of juvenile animals^3,34,50^ and reduced post junctional responses to nerve stimulation in the stomach^51^. Activation of Ano1 in ICC is due to Ca^2+^ release from intracellular ER stores^6^, and Ca^2+^ release and the spontaneous transient inward currents (STICs) resulting from activation of Ano1 channels are inhibited by blocking the refilling of the ER with the SERCA pump inhibitor, thapsigargin^6,30,31^, or by depleting ER stores by inhibiting SOCE in ICC^38^. We reasoned that Ano1 antagonists are a useful tool for determining the role of Ano1 channels, and thus mechanisms intrinsic to ICC, in GI motor patterns. Since Ano1 channels in ICC are activated by Ca^2+^ release we also reasoned that interfering with Ca^2+^ release by inhibiting the SERCA pump or SOCE may also indicate an ICC-dependent mechanism, as excitation-contraction coupling in SMCs is primarily dependent upon Ca^2+^ entry via voltage-dependent Ca^2+^ channels and not Ca^2+^ release mechanisms^52^. We found that Ca^2+^ handling antagonists, thapsigargin and the Orai channel inhibitor central to SOCE, GSK 7975 A, significantly reduced the colonic contractions unleashed by TTX, L-NNA and ODQ. These observations are consistent with the idea that NO-dependent inhibition of Ca^2+^ release in ICC reduces activation of Ano1 and this causes reduced SMC excitability and contraction.

At present, how NO interferes with Ca^2+^ handling in ICC is poorly understood. Certain phosphorylation-dependent mechanisms can modulate the activation of ER Ca^2+^ release channels, such as IP_3_Rs^53^. Either cGMP-dependent protein kinase G (PKG)^54,55^ phosphorylation of IP_3_R or a downstream modulator of NO signaling, such as IP_3_R-associated cGMP-kinase substrate (IRAG; encoded by *Mrvi1*)^56^ might affect Ca^2+^ release in response to nitrergic stimulation. PKG, as well as protein kinase A, have been shown to limit Ca^2+^ release in myocytes and endothelial cells of the vasculature^57^ and in interstitial cells from the rabbit urethra^55,58^. IRAG has been shown to be involved in cGMP-dependent regulation of Ca^2+^ release in model cells^56^ and in NO-mediated relaxation of human colon^59^. IP_3_Rs are known to facilitate Ca^2+^ release in ICC^6,30,31^, thus its regulation would be expected to modulate Ca^2+^ transients in ICC. More in depth studies of the downstream coupling between NO-dependent cGMP production and Ca^2+^ release in ICC should be a goal of future studies.

While our data indicate that ICC bear significant responsibility for mediation of tonic inhibition, other cells that are part of the SIP syncytium must be considered for contributions. PDGFRα^+^ cells express P_2_Y_1_ receptors and SK3 channels in abundance and appear to mediate purinergic responses in the colon through P_2_Y_1_ receptor pathways^9,12,60^. These cells also express signaling molecules, such as the subunits of guanylate cyclase and PKG^43,61^. PDGFRα^+^ cells via purinergic inhibition are not likely to be involved in tonic inhibition in the proximal colon, because a P_2_Y_1_ receptor antagonist, MRS 2500, did not increase contractions. The question of mediation of nitrergic tonic inhibition is more difficult, but peripheral information suggests that PDGFRα^+^ cells are unlikely to contribute to nitrergic inhibition in the colon. NO causes inhibition of Ca^2+^ release in ICC (Fig. 8), but if a similar phenomenon occurred in PDGFRα^+^ cells, the obligatory response would be decreased activation of SK3 channels, depolarization and increased SMC excitability. Contributions of nitrergic responses in SMCs are also possible, and results of experiments with genetic knock-down of soluble guanylyl cyclase (sGC) in either SMCs or ICC suggested that both ICC and SMCs are responsible for the effects of NO on contractile frequency in the proximal colon^62^. The mechanism for increasing contractile frequency in the sGC-SMC knock-down was unexplained, but a possible mechanism for increasing the force of contractions in SMCs directly might be changes in Ca^2+^ sensitivity of the contractile apparatus. Ca^2+^ sensitivity in SMCs is regulated by cyclic nucleotides, such that cGMP-dependent mechanisms would tend to reduce the force of contractions for a given level of Ca^2+^ in SMCs. In general, the response to Ano1 antagonists in our study, an effect on channels in ICC, was a reduction in contractile frequency. One would expect that effects on Ca^2+^ sensitivity would be changes in contractile amplitude, a response intrinsic to SMCs, without effect on contractile frequency.

Benzbromarone has been shown to block Ano1 channels in several previous studies^63–65^, and Ani9 (1 μM), a newer Ano1 antagonist, was shown to block currents due to expression of human ANO1(abc splice variant) expressed in Fisher rat thyroid (FRT) cells (i.e. IC50 of ~110 nM)^35^. However, the specter of off-target effects is always of concern with antagonists of Ca^2+^-activated Cl^−^ channels. In fact several Ano1 antagonists, said to be selective, have been shown to inhibit L-type Ca^2+^ channels. Such an effect could provide an alternative explanation to our findings because colonic contractions might also be inhibited by blocking L-type Ca^2+^ channels and reducing Ca^2+^ influx into SMCs (e.g. Figs 2 and 3). In fact we discovered significant off-target effects with benzbromarone, even at the low concentrations utilized in these studies, because 1 & 3 μM of this antagonist inhibited KCl contractions significantly, suggesting blocking effects on L-type Ca^2+^ channels. Ani9 (3 μM) also reduced KCl contractions by ~30% (Fig. 7D), but at 1 μM Ani9 had no effect on elevated [K^+^]_o_ contractions. At this concentration, Ani9 had significant effects on the contractions enhanced by TTX (Figs 2B,D and S2B,F), L-NNA (Fig. 3B,D) and ODQ (Fig. 3F,H), making this drug suitable to test the hypothesis that suppression of Ano1 channels in ICC is a viable mechanism of tonic inhibition in the colon.

In summary, tonic inhibition, a well-described and important motility mechanism that suppresses the excitability and contractile activity of colonic SMCs, is mediated in part by ICC by suppression of Ca^2+^ release from stores and reduction of Cl^−^ efflux (i.e. inward current) through Ano1 channels (Fig. 8). Suppression of nitrergic neural input to muscles by TTX or L-NNA or inhibition of post-junctional transduction of NO by ODQ enhanced Ca^2+^ transients in ICC, which are known to be linked to activation of Ano1 channels^6^. Inward currents through Ano1 channels in ICC have a depolarizing effect on the SIP syncytium, so reduction in Ano1 current serves to reduce SMC excitability. Our data also indicate that the normal patterning of colonic contractions is tightly related to periodic activation of Ano1 channels in ICC, as frequency and amplitude of contractions were reduced by a concentration of Ano1 antagonist (Ani9; 1μM) that suppresses Ano1 current nearly 100%^35^. The results of this study demonstrate a novel function of ICC in regulating the motility pattern known as tonic inhibition in the colon.

## Methods

### Ethical approval

Animals used and the protocols performed in this study were in accordance with the National Institutes of Health Guide for the Care and Use of Laboratory Animals. All procedures were approved by the Institutional Animal Use and Care Committee at the University of Nevada, Reno.

### Tissue preparation

For contractile studies, wildtype mice (C57BL/6; 10–14 weeks old; Jackson Laboratory, Bar Harbor, MN, USA) were anaesthetized by isoflurane inhalation (Baxter, Deerfield, IL, USA) and then sacrificed by cervical dislocation. The GI tract en masse was removed through an abdominal incision and transferred to a dish containing Krebs-Ringer-bicarbonate (KRB) solution of the following composition (in mM): NaCl 118.5, KCl 4.7, CaCl_2_ 2.5, MgCl_2_ 1.2, NaHCO_3_ 23.8, KH_2_PO_4_ 1.2, dextrose 11.0. This solution had a pH of 7.4 at 37 °C when bubbled to equilibrium with 95% O2–5% CO_2_. Colons were removed from mice, pinned down in a Sylgard-lined dish and opened longitudinally. Mucosa and submucosa were peeled away leaving the *tunica muscularis* intact.

For ICC Ca^2+^ imaging experiments, we utilized a genetically-encoded Ca^2+^ indicator, GCaMP6f, expressed exclusively in ICC using the Cre-Lox P technique, where an inducible Cre Recombinase (induced by tamoxifen injection) was driven by an ICC specific promoter (tyrosine kinase receptor *Kit*). Ai95 (RCL-GCaMP6f)-D (GCaMP6f mice) were purchased from the Jackson Laboratory (Bar Harbor, MN, USA). c-Kit^+/Cre-ERT2^ (Kit-Cre mice) were gifted from Dr. Dieter Saur of the Technical University Munich, Germany. GCaMP6f mice were crossed with Kit-Cre mice and the resultant offspring are referred to as Kit-Cre-GCaMP6f mice throughout the manuscript. These animals were injected with tamoxifen at ages of 6–8 weeks to induce Cre Recombinase and subsequent GCaMP6f expression. Tamoxifen (Sigma T5648; 80 mg) was dissolved in 800 μL of ethanol (Pharmco-Aaper 200 Proof - Absolute, Anhydrous) by vigorous vortexing for 20 minutes. Then, 3.2 ml of Safflower (generic) was added to create solutions of 20 mg/ml, which were then sonicated for 30 minutes prior to injection. Mice were injected (Intraperitoneal injection; IP) with 0.1 ml of tamoxifen solution (2 mg tamoxifen) for three consecutive days. Mice were used 10 days after the first injection and the subsequent expression of GCaMP6f was confirmed by genotyping. Kit-Cre-GCaMP6f mice were sacrificed and flat sheets of proximal colon, with the mucosa removed were prepared in the same manner as wildtype animals.

### Contractile recordings

Muscle strips (5–8 mm in length and ~2 mm in diameter) were cut parallel to the circular muscle fibers from the proximal colon, attached to a stable mount and to a Gould strain gauge and immersed in jacketed 15 ml tissue baths. The muscles were continuously oxygenated and maintained in KRB solution at 37 °C. Tissues were stretched to an initial tension of 5 mN, and experiments began after 60 mins of equilibration. Tissues were washed with fresh, oxygenated KRB solution every 20 mins. Isometric contractions were recorded using AcqKnowledge software (3.9.1; Biopac Systems, Goleta, CA).

### Calcium imaging

The proximal colon was pinned with the circular muscle (CM) layer facing upward to the base of a 5 ml chamber (60 mm diameter) coated with Sylgard. The muscles were continuously perfused with warmed KRB solution at 37 °C for an equilibration period of 1 hour before experimentation. Following this equilibration period, imaging of Ca^2+^ transients in ICC *in situ* was acquired using an Eclipse E600FN microscope (Nikon Inc., Melville, NY, USA) equipped with a 60 × 1.0 CFI Fluor lens (Nikon instruments INC, NY, USA). GCaMP6f was excited at 488 nm (T.I.L.L. Polychrome IV, Grafelfing, Germany), as previously described^37,66^. The pixel size using this acquisition configuration was 0.225 µm. Image sequences were collected at 33 fps with TILLvisION software (T.I.L.L. Photonics GmbH, Grafelfing, Germany). Ca^2+^ imaging experiments were performed in the presence of nicardipine (100 nM) to minimize movement artefacts resulting from contractions. For experiments with pharmacological interventions, a control period of 30 sec was recorded and then KRB solution containing the appropriate concentration of drug was perfused into the bath for 15 mins to allow for full tissue penetration before acquiring another 30 sec recording to ascertain the effects of the drug on ICC.

### Data analysis

Isometric contractions were analyzed using Clampfit software (Axon Instruments). Area under the curve (AUC) measurements from control periods were normalized to 1.0, and changes in AUC were normalized as a fold changes from the control values. AUC under control conditions was tabulated during the 10 min immediately before addition of the drugs, and responses to the drugs were tabulated from the period 10–20 min after adding the drug. For imaging files, Ca^2+^ transients were quantified with spatio-temporal maps as described previously^67^. Briefly, movies of Ca^2+^ activity in ICC were converted to a stack of TIFF (tagged image file format) images and spatio-temporal maps (STMs) of Ca^2+^ activity were generated in individual ICC within a FOV using Image J (version1.52a, National Institutes of Health, MD, USA, http://rsbweb.nih.gov/ij). The basal Ca^2+^ fluorescence was acquired from regions of the cell displaying the most uniform and least intense fluorescence (F_0_), the fluorescence value throughout the cell was then divided by the F_0_ value to calibrate the STM for amplitude of Ca^2+^ events as F/F_0_. Ca^2+^ transient frequency in ICC was expressed as the number of events fired per minute (min^−1^). The amplitude of Ca^2+^ transients was expressed as ΔF/F_0_, the duration of Ca^2+^ transients was expressed as full duration at half maximum amplitude (FDHM) and the spatial spread of Ca^2+^ transients was expressed as μm of cell propagated per Ca^2+^ transient. In experiments where drugs were applied to ICC activity, quantification of Ca^2+^ transient parameters in control and in the presence of a drug would be analyzed in 3–5 paired representative cells in a FOV per animal used in experimentation.

### Statistics

Throughout the manuscript, n values represent the number of muscle strips or cells in a dataset, and all datasets represent data from a minimum of 3 animals. Data were analyzed for statistical significance using Prism (Version 6.0c, GraphPad Software Inc., San Diego, CA, USA), and values are expressed as mean ± SEM. Statistical analyses used a paired students t test or a two way ANOVA with a post hoc Tukey multiple comparison test as appropriate. P values < 0.05 were considered to be statistically significant.

### Drugs

Tetrodotoxin (TTX), 2-(4-chloro-2-methylphenoxy)-N-[(2-methoxyphenyl) methylideneamino]-acetamide (Ani-9), Benzbromarone, Nω-nitro-L-arginine (L-NNA), 1*H*-[1,2,4] Oxadiazolo [4,3-*a*] quinoxalin-1-one (ODQ), and Thapsigargin were purchased from Tocris Bioscience (Ellisville, Missouri, USA). GSK 7975 A was purchased from Aobious (Gloucester, MA).

## Supplementary information

Supplementary Information

## Data Availability

The datasets generated during and/or analysed during the current study are available from the corresponding author on reasonable request.
