# Supplementary material for: Tonic inhibition of murine proximal colon is due to nitrergic suppression of Ca2+ signaling in interstitial cells of Cajal
Source: Sci Rep. 2019 Mar 13;9:4402. doi: 10.1038/s41598-019-39729-7 (PMC6416298; doi:10.1038/s41598-019-39729-7)
Supplement: Supplementary file 1 — Supplementary Information [file 41598_2019_39729_MOESM1_ESM.pdf]

# SUPPLEMENTAL INFORMATION

## Tonic inhibition of murine proximal colon is due to nitrergic suppression of $Ca^{2+}$ signaling in interstitial cells of Cajal

Bernard T. Drumm*[1], Benjamin E. Rembetski[1], Salah A. Baker[1] & Kenton M. Sanders[1]

[1]Department of Physiology & Cell Biology,
University of Nevada, Reno School of Medicine,
Reno NV, U.S.A. 89557

***Corresponding author:** Bernard T. Drumm, Department of Physiology & Cell Biology, University of Nevada, Reno School of Medicine, MS 352, Reno, NV, 89557, USA. Phone: (775) 685-0975. Fax (775) 784-6903. Email: bdrumm@med.unr.edu

Fig.S1

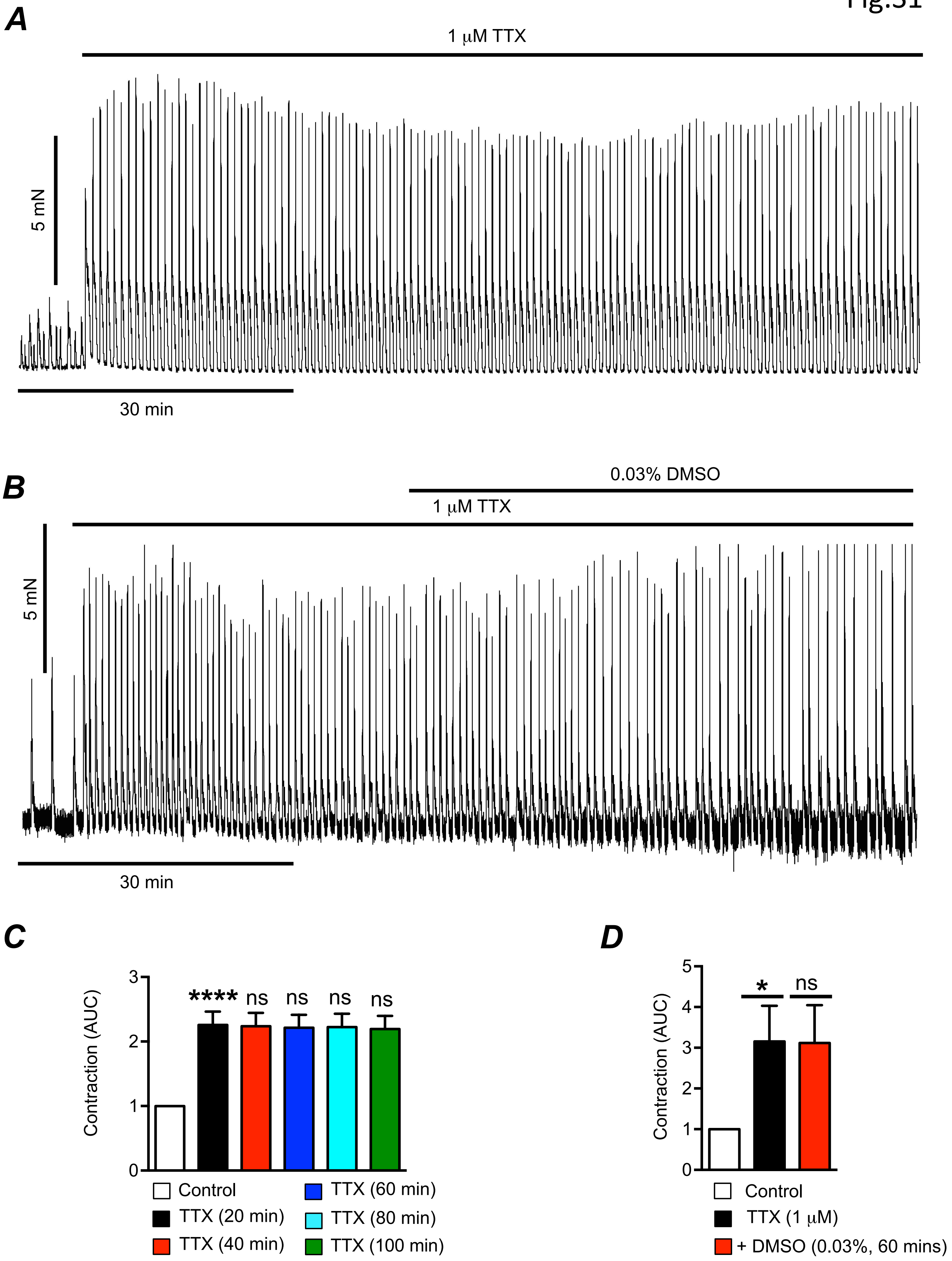
A
1 μM TTX
5 mN
30 min
B
0.03% DMSO
1 μM TTX
5 mN
30 min
C
Contraction (AUC)
0
1
2
3
****
ns
ns
ns
ns
Control
TTX (20 min)
TTX (40 min)
TTX (60 min)
TTX (80 min)
TTX (100 min)
D
Contraction (AUC)
0
1
2
3
4
5
*
ns
Control
TTX (1 μM)
+ DMSO (0.03%, 60 mins)

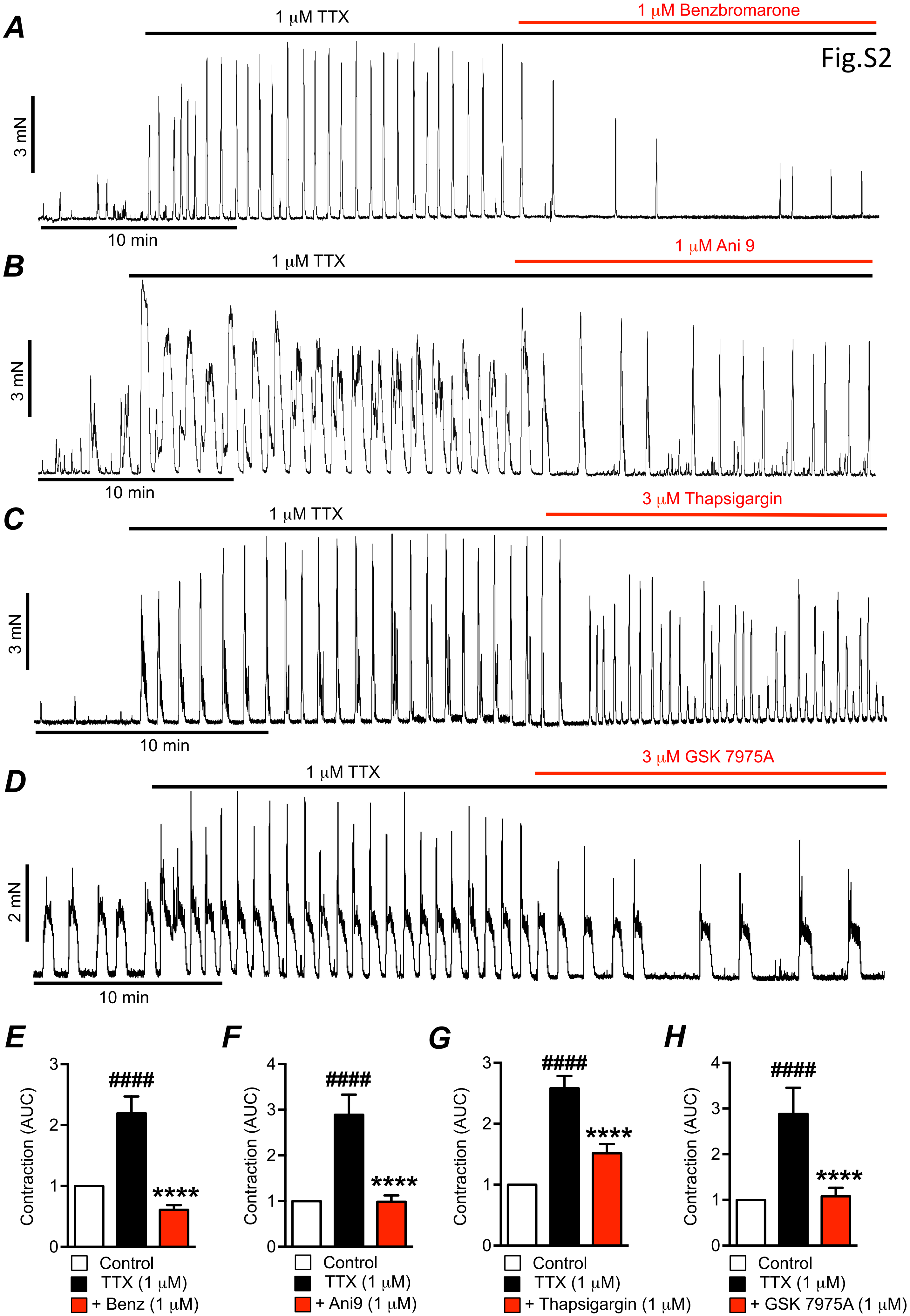
Fig.S2
A
1 μM TTX
1 μM Benzbromarone
3 mN
10 min
B
1 μM TTX
1 μM Ani 9
3 mN
10 min
C
1 μM TTX
3 μM Thapsigargin
3 mN
10 min
D
1 μM TTX
3 μM GSK 7975A
2 mN
10 min
E
Contraction (AUC)
####
****
Control
TTX (1 μM)
+ Benz (1 μM)
F
Contraction (AUC)
####
****
Control
TTX (1 μM)
+ Ani9 (1 μM)
G
Contraction (AUC)
####
****
Control
TTX (1 μM)
+ Thapsigargin (1 μM)
H
Contraction (AUC)
####
****
Control
TTX (1 μM)
+ GSK 7975A (1 μM)

**Supplemental Figure 1: Time and vehicle controls for the effect of Ano1 blockers.** ***A*** Representative contractile trace showing the effect of TTX (1 μM) on proximal colon contractions over 100 mins of recording. ***B*** Representative contractile trace showing the effect of 0.03 % DMSO on the excitatory effects of TTX (1 μM) on proximal colon contractions over 60 mins of recording. ***C*** Summarized data showing quantification of colonic contractions every 20 mins in the continued presence of TTX (1 μM) (n=9). ***D*** Summarized data showing quantification of colonic contractions in the continued presence of TTX (1 μM) after a 60 min application of 0.03 % DMSO (n=9). ns = P>0.05 compared to TTX, * = P<0.05 compared to control, **** = P<0.0001 compared to control.

**Supplemental Figure 2: The acute effect of Ano1 channel and $Ca^{2+}$ handling inhibitors on the excitatory response of TTX.** ***A*** Representative contractile trace showing the effect of benzbromarone (1 μM) on the excitatory response of TTX (1 μM) on proximal colon contractions. ***B*** Representative contractile trace showing the effect of Ani 9 (1 μM) on the excitatory response of TTX (1 μM) on proximal colon contractions. ***C*** Representative contractile trace showing the effect of thapsigargin (3 μM) on the excitatory response of TTX (1 μM) on proximal colon contractions. ***D*** Representative contractile trace showing the effect of GSK 7975A (3 μM) on the excitatory response of TTX (1 μM) on proximal colon contractions. ***E*** Summarized data showing the effect of benzbromarone (1 μM) on the excitatory response of TTX (1 μM) on proximal colon contractions (n=23). ***F*** Summarized data showing the effect of Ani 9 (1 μM) on the excitatory response of TTX (1 μM) on proximal colon contractions (n=14). ***G*** Summarized data showing the effect of thapsigargin (3 μM) on the excitatory response of TTX (1 μM) on proximal colon contractions (n=27). ***H*** Summarized data showing the effect of GSK 7975A (3 μM) on the excitatory response of TTX (1 μM) on proximal colon contractions (n=13). ### = P<0.001 compared to control, #### = P<0.0001 compared to control, **** = P<0.0001 compared to TTX.
